# Towards standardization of ^18^F-FET PET imaging: do we need a consistent method of background activity assessment?

**DOI:** 10.1186/s13550-017-0295-y

**Published:** 2017-05-30

**Authors:** Marcus Unterrainer, Franziska Vettermann, Matthias Brendel, Adrien Holzgreve, Michael Lifschitz, Matthias Zähringer, Bogdana Suchorska, Vera Wenter, Ben M. Illigens, Peter Bartenstein, Nathalie L. Albert

**Affiliations:** 1 Department of Nuclear Medicine, LMU Munich, Marchioninistr. 15, 81377 Munich, Germany; 2 Center for Clinical Research and Management Education, Division of Health Care Sciences, Dresden International University, Dresden, Germany; 3 Department of Neurosurgery, LMU Munich, Munich, Germany; 4 Department of Neurology, Beth Israel Deaconess Medical Center, Harvard Medical School, Boston, MA USA

**Keywords:** ^18^F-FET PET, Background, Brain tumor, Glioma

## Abstract

**Background:**

PET with O-(2-^18^F-fluoroethyl)-L-tyrosine (^18^F-FET) has reached increasing clinical significance for patients with brain neoplasms. For quantification of standard PET-derived parameters such as the tumor-to-background ratio, the background activity is assessed using a region of interest (ROI) or volume of interest (VOI) in unaffected brain tissue. However, there is no standardized approach regarding the assessment of the background reference. Therefore, we evaluated the intra- and inter-reader variability of commonly applied approaches for clinical ^18^F-FET PET reading.

The background activity of 20 ^18^F-FET PET scans was independently evaluated by 6 readers using a (i) simple 2D-ROI, (ii) spherical VOI with 3.0 cm diameter, and (iii) VOI consisting of crescent-shaped ROIs; each in the contralateral, non-affected hemisphere including white and gray matter in line with the European Association of Nuclear Medicine (EANM) and German guidelines. To assess intra-reader variability, each scan was evaluated 10 times by each reader. The coefficient of variation (CoV) was assessed for determination of intra- and inter-reader variability. In a second step, the best method was refined by instructions for a guided background activity assessment and validated by 10 further scans.

**Results:**

Compared to the other approaches, the crescent-shaped VOIs revealed most stable results with the lowest intra-reader variabilities (median CoV 1.52%, spherical VOI 4.20%, 2D-ROI 3.69%; *p* < 0.001) and inter-reader variabilities (median CoV 2.14%, spherical VOI 4.02%, 2D-ROI 3.83%; *p* = 0.001). Using the guided background assessment, both intra-reader variabilities (median CoV 1.10%) and inter-reader variabilities (median CoV 1.19%) could be reduced even more.

**Conclusions:**

The commonly applied methods for background activity assessment show different variability which might hamper ^18^F-FET PET quantification and comparability in multicenter settings. The proposed background activity assessment using a (guided) crescent-shaped VOI allows minimization of both intra- and inter-reader variability and might facilitate comprehensive methodological standardization of amino acid PET which is of interest in the light of the anticipated EANM technical guidelines.

## Background

PET with O-(2-^18^F-fluoroethyl)-L-tyrosine (^18^F-FET) has reached increasing clinical significance for the workup of patients suffering from brain neoplasms as an additional and highly valuable imaging tool. As recently emphasized by the RANO working group [1], clinical applications of ^18^F-FET PET are represented by, e.g., treatment planning [2], patient monitoring [3], prognostication at initial diagnosis [4, 5], and evaluation of pseudoprogression [6]. Particularly, PET parameters such as the maximal tumor-to-background-ratio (TBR_max_) are used in clinical routine, e.g., for response assessment of alkylating and antiangiogenic agents [7–9] or differentiation of viable tumor from treatment-related changes [10–13].

For evaluation of ^18^F-FET PET parameters, a background-activity reference in unaffected brain tissue is used to enable intra- and inter-individual comparability of PET results. The European Association of Nuclear Medicine (EANM) guideline for brain tumor imaging stated “Interpretation of quantitative results is based on the comparison of tumor-to-background uptake ratio,” and although the guideline pointed to a potential source of error by “small regional differences in uptake in normal brain, emphasizing the need for careful choice of an appropriate reference region” [14], there is no procedural recommendation regarding the method of background assessment. Therefore, several different and inconsistent approaches for background assessment are used in the current literature and in the clinical routine: one approach uses a region of interest (ROI) in the contralateral hemisphere including white and gray matter [15, 16], which is in line with the German guideline for amino acid imaging, which stated that a ROI should be placed in unaffected contralateral brain tissue, “e.g., with a diameter of 50 mm” [17]. Other approaches apply volumes-of-interest (VOI) including gray and white matter, i.e., a spherical VOI with a diameter of 30 mm [18, 19] or a VOI consisting of crescent-shaped ROIs [13, 20]. Although first suggestions were made regarding a software-based assessment using [^11^C]-methionine PET previously [21], there is still no standardized and consistent procedure used in the clinical routine.

Hence, we intended to elucidate the effects of different approaches for background activity assessment and to evaluate simple and clinically applicable methods of background activity assessment for ^18^F-FET PET imaging regarding their inter- and intra-reader variability in the light of an emphasized comprehensive standardization of amino acid PET.

## Methods

### PET acquisition and data evaluation

Dynamic ^18^F-FET PET scans were acquired with an ECAT Exact HR+ scanner (Siemens, Erlangen, Germany) according to standard protocols [22], after a fasting period of at least 6 h prior to PET scanning. After a 15-min transmission scan with a ^68^Ge rotating rod source, approximately 180 MBq of ^18^F-FET were injected as an intravenous bolus. Afterwards, the 40-min dynamic emission recording in 3-D mode consisting of 16 frames (7 × 10 and 3 × 30 s; 1 × 2, 3 × 5, and 2 × 10 min) was started. Images corrected for attenuation and scatter were reconstructed by filtered back-projection using a 5-mm Hann filter. For conventional semi-quantitative evaluation, the maximal tumoral ^18^F-FET uptake (SUV_max_) was determined on a summation image (20–40 min after injection). The biological-tumor-volume (BTV) was estimated by semiautomatic calculation of a VOI using a threshold of TBR ≥ 1.6, previously proposed as optimal threshold for differentiation of tumor and surrounding healthy tissue [23]. ^18^F-FET PET was evaluated on a Hermes workstation (Hermes Medical Solutions, Stockholm, Sweden) as described previously [22].

### ^18^F-FET PET scans

Twenty ^18^F-FET PET scans of patients with histologically proven glioma were randomly selected for background activity assessment. In a second step, a randomly selected control cohort including 10 additional ^18^F-FET PET scans was used to assess the guided background assessment on an intra-individual and inter-individual basis. All patients gave written consent to undergo ^18^F-FET PET as part of the clinical routine. The retrospective study was approved by the local ethical review board.

### Readers and background activity assessment

Six readers performed an evaluation of the following three methods of background activity assessment in all 20 scans according to the following instructions:

1. Simple ROI: A single 2D-ROI in the contralateral hemisphere including white and gray matter (e.g., 50 mm diameter), as proposed in the German guideline [17].
2. Spherical VOI: A spherical VOI with a diameter of 30 mm in the contralateral hemisphere including white and gray matter, as published previously [18, 19].
3. Crescent-shaped VOI: A merged VOI consisting of six crescent-shaped ROIs in the contralateral hemisphere including white and gray matter as published previously [13, 20].

**Fig. 1:**
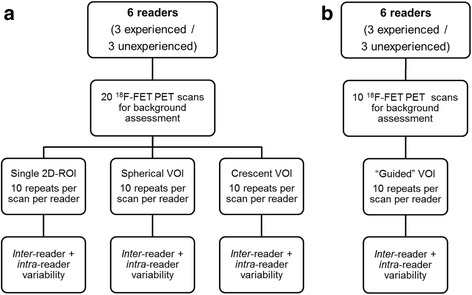
Procedural outline. **a** Evaluation of three commonly used methods for background assessment, **b** evaluation of the best method of **a** with additional guidance through instructions

**Fig. 2:**
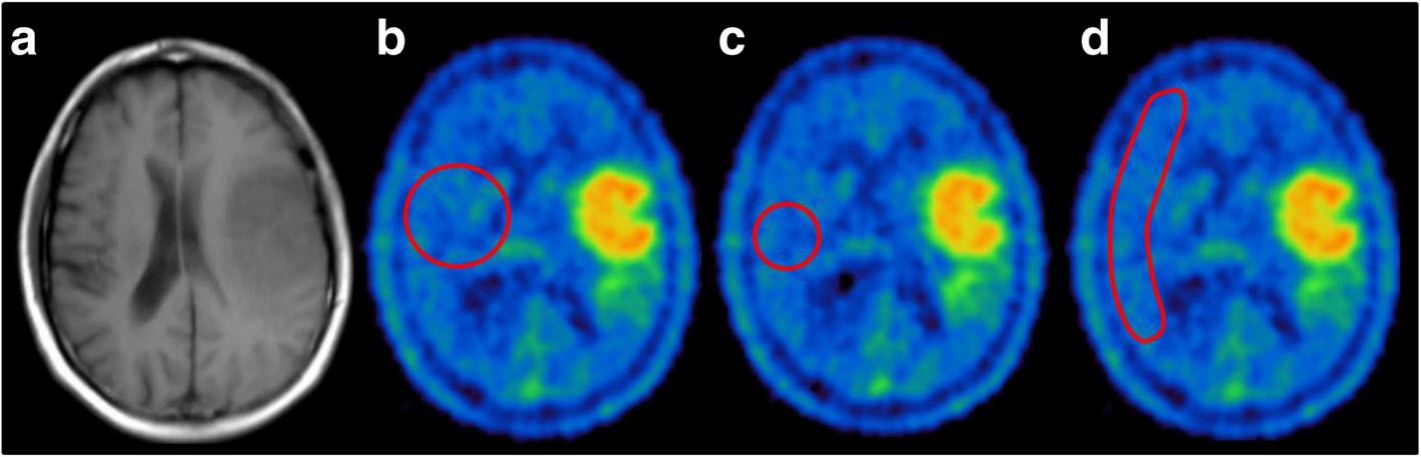
Examples of the different methods of background assessment. **a** T1 MRI for morphological correlation. The three different methods of background activity assessment are featured using **b** a 2D-ROI in the contralateral hemisphere with 5 cm diameter as example (as suggested in the German guideline), **c** a spherical VOI with 3 cm diameter, and **d** a crescent-shaped VOI approach

To evaluate the intra-reader variability of each method, each reader independently evaluated the row of 20 scans for 10 times (a methodologic outline is shown in Fig. 1, examples of the different approaches are displayed in Fig. 2). Of the 6 readers, 3 were experienced (M.U., F.V., M.B.; each with >500 clinical ^18^F-FET PET reads) and 3 unexperienced (A.H., M.L., M.Z.) in the clinical reading of ^18^F-FET PET scans.

**Fig. 3:**
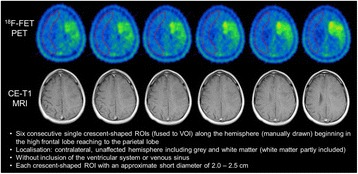
Instruction for the guided, crescent-shaped VOI approach

In a second step, the best method of the first study part was applied in additional 10 control scans. In analogy to the first evaluation, the additional scans were analyzed by each reader independently and repeatedly (10 times). As amendment, the readers were prompted to use standardized instructions for guidance and precision of background assessment in order to reduce inter-individual variability (see Fig. 3).

### Statistics

Statistical analyses were performed with IBM^®^ SPSS^®^ Statistics, Version 23. As standardized measure of dispersion, the “coefficient of variation” (CoV), defined as ratio of the standard deviation to the mean, was applied to assess the intra-reader variability (individual CoV of a particular scan) as well as the inter-reader variability (CoV of the mean SUV values of the six readers regarding a particular scan). Normal distribution was assessed using the Shapiro-Wilk test. The intra- and inter- individual CoVs of the different reference approaches were compared using the non-parametric Friedman-/Kruskal-Wallis test and the paired/unpaired Mann-Whitney *U* test, respectively. Statistical significance was defined as two-tailed *p* values below 0.05.

## Results

### Simple ROI

When applied in all readers, the use of a simple ROI in the contralateral hemisphere showed a median intra-individual variability of a CoV of 3.69% (range 1.90–7.05%) with a homogenous distribution throughout all 6 readers. When comparing the individual mean SUV-measurements in all 20 scans, a median variability (inter-reader CoV) of 3.83% (range 1.80–7.46%) could be observed (see Table 1).

**Table 1:** Intra- and inter-reader coefficients of variation using VOI/ROIs [median (range)]

	Simple ROI	Spherical VOI	Crescent VOI	Friedman test
Intra-reader variabilities				
Overall group	3.69% (1.90–7.05%)	4.20% (1.74–8.30%)	1.52% (0.48–3.78%)	*p* < 0.001
Reader #1	4.03% (1.90–7.05%)	4.83% (2.58–8.30%)	1.04% (0.55–3.00%)	*p* < 0.001
Reader #2	5.16% (2.37–6.23%)	4.38% (2.79–5.98%)	1.49% (0.97–2.25%)	*p* < 0.001
Reader #3	2.86% (2.07–5.38%)	2.72% (1.74–3.53%)	1.19% (0.48–2.27%)	*p* < 0.001
Reader #4	2.99% (2.23–4.20%)	3.97% (2.59–6.68%)	2.06% (1.11–2.75%)	*p* < 0.001
Reader #5	2.62% (1.95–4.06%)	3.09% (2.02–5.04%)	1.05% (0.39–2.42%)	*p* < 0.001
Reader #6	4.74% (2.42–6.96%)	5.46% (3.04–6.57%)	1.97% (1.07–3.78%)	*p* < 0.001
*Inter*-reader variabilities				
Overall group	3.83% (1.80–7.46%)	4.02% (1.47–6.32%)	2.14% (1.05–7.23%)	*p* = 0.001

There was no statistically different intra-reader variability between experienced and unexperienced readers (3.15% (1.95–6.23%) vs. 3.69% (1.90–7.05%), *p* = 0.087). Experienced readers showed a significantly smaller variability in terms of inter-reader variability (1.63% (0.05–5.00%) vs. 4.37% (0.59–5.37%), *p* = 0.001).

### Spherical VOI

The approach using a spherical VOI showed comparable intra-reader variabilities with a median CoV of 4.20%, but provided an even broader CoV range (1.74–8.30%) (see Table 1). When comparing the mean SUV values between the 6 readers, a median inter-reader CoV of 4.02% with a relatively broad range throughout the 20 included scans could be observed (range 1.47–6.32%).

Experienced readers provided a significantly smaller intra-reader variability (median CoV 3.22% (1.74–5.98%) vs. 4.63% (2.58–8.30%); *p* < 0.001), whereas no significant difference (2.16% (0.61–5.63%) vs. 2.71% (0.59–5.37%), *p* = 0.970) in terms of inter-reader variability between experienced and unexperienced could be observed.

### Crescent VOI

The use of crescent-shaped VOIs for background activity assessment revealed a median intra-individual CoV of 1.52% (range 0.48–3.78%). Although the median inter-rater CoV was low with a value of 2.14%, there were some scans with outlining inter-reader variabilities of up to 7% (range 1.05–7.23%).

Experienced readers revealed a significantly smaller intra-reader variability (median CoV 1.20% (0.39–2.42%) vs. 1.81% (0.55–3.78%); *p* < 0.001), whereas no significant difference in terms of inter-reader variability could be observed between experienced and unexperienced readers (median CoV 1.36% (0–6.13%) vs. 1.97% (0.63–9.57%), *p* = 0.537).

### Comparison of background assessment methods

All six readers obtained the most stable background activity values at the lowest median intra-reader CoV by the use of the crescent-shaped VOI when compared to the simple ROI (*p* < 0.001 in each reader) or the spherical VOI (*p* < 0.001 in each reader). Additionally, the crescent-VOI approach revealed the significantly lowest inter-reader variability as well when compared to the other methods (*p* = 0.001; see also Table 1).

### Background activity assessment using the guided crescent-shaped VOI

In the guided approach using the clearly defined instructions, the inter-reader variability could be significantly reduced to a median CoV of 1.19% (range 0.84–1.89%, *p* = 0.001) with a considerably smaller range compared to the unguided approach (see Table 2). Furthermore, the median intra-reader variability was 1.10% (range 0.52–2.36%), which was significantly lower (*p* < 0.001) than the unguided crescent-VOI approach.

**Table 2:** Intra- and inter-reader coefficients of variation using the guided VOI [median (range)]

Intra-reader variabilities	
Overall group	1.10% (0.52–2.36%)
Reader #1	1.06% (0.74–1.77%)
Reader #2	0.81% (0.66–0.99%)
Reader #3	1.06% (0.74–1.31%)
Reader #4	1.73% (1.13–2.36%)
Reader #5	1.00% (0.70–1.75%)
Reader #6	0.93% (0.52–1.57%)
Inter-reader variabilities	
Overall group	1.19% (0.84–1.89%)

No statistically different median CoV between experienced and unexperienced readers (1.01% (0.70–1.75%) vs. 1.20% (0.52–2.36%), *p* = 0.060) could be detected using the guided VOI approach regarding intra-reader variability. Nonetheless, experienced readers showed a smaller variability in terms of inter-reader variability (0.63% (0–1.39%) vs 1.42% (0–2.88%), *p* = 0.037) when compared to the unexperienced readers.

## Discussion

Complementary to MRI, ^18^F-FET PET has gained clinical importance for the diagnostic workup of brain tumor patients in all disease stages, as recently recommended by the RANO and EANO working group [1]. However, the methods of PET evaluation are not yet standardized. In particular, the approaches for background activity assessment are variable, although background assessment is essential for the evaluation of TBR values as well as quantification of biological tumor volume. For the implementation of ^18^F-FET PET into clinical trials, a methodological PET standardization is urgently needed to enable more precise and reproducible ^18^F-FET PET quantification.

Looking at the present data it can be stated that the different approaches under investigation show a certain variability on an intra-individual as well as on an inter-individual basis with expectable background SUV changes up to ±8% (which means a notable dispersion of up to 16%) and might therefore be considered as potential source of methodological error besides known influencing factors such as different reconstruction algorithms and diverging PET scanners.

When evaluating the different background assessment approaches, the use of a single ROI showed a relatively high intra- and inter-reader variability; this might be due to the assessment of a relatively small regional part of unaffected brain only (ROI vs. VOI in the other approaches) as well as unprecise description regarding shape and spatial localization, as ambiguously stated in the German guideline for amino-acid PET (“[…] a larger background ROI in the contralateral and unaffected hemisphere including gray and white matter (diameter, e.g., 50 mm)”) [17].

Although the application of a spherical VOI with a fixed diameter of 3 cm represents a uniform approach without individual changes regarding the shape of the VOI, this approach likewise showed a relatively high variability; the rigid shape did not necessarily lead to high stability of SUV measurement since areas inappropriate for background assessment might be included inevitably due to individual morphologic properties, e.g., the ventricular system.

In comparison to these two approaches, the use of crescent-shaped VOIs provided both the lowest intra-reader and inter-reader variability. An advantage of this approach might be the possible adaption of the individual morphologic properties. Nonetheless, some single outlining scans with an inter-reader CoV up to 7% occurred in our analysis, most likely due to a differing localization of the six sequential crescent-shaped ROIs in different brain areas with slightly different background activities throughout the six readers. This can be stated since the variabilities were very small within single readers, leading to the assumption that every single reader had a constant method of manual VOI definition. Therefore, we consequently intended to guide the manual defining of the crescent-shaped VOI to further reduce the inter-reader variability using the instructions described in the “Methods” section. These instructions intended to provide a high standardization in terms of shape and localization of the applied VOIs. Indeed, this approach showed the lowest variability between the readers with a median inter-reader CoV of 1.19% (range 0.84–1.89%) and therefore a maximum expectable dispersion <4% regarding the background SUV. Interestingly, this approach could additionally reduce the individual intra-reader variabilities when compared to the “unguided” crescent-VOI approach (1.10% (range 0.52–2.36%) vs. 1.52% (range 0.48–3.78%)). Besides, the possibility of an adaption of the VOI shape according to individual morphologic properties, an explanation for the low variability of the crescent-shaped VOI approach might be that the summation of six crescent-shaped ROIs led to relatively large volumes, which could also contribute to the higher stability. Furthermore, the crescent-shaped VOI is characterized by a balanced inclusion of gray and white matter, while the proportion and composition of included structures might be more variable depending on the exact positioning of a ROI/VOI with a fix shape.

**Fig. 4:**
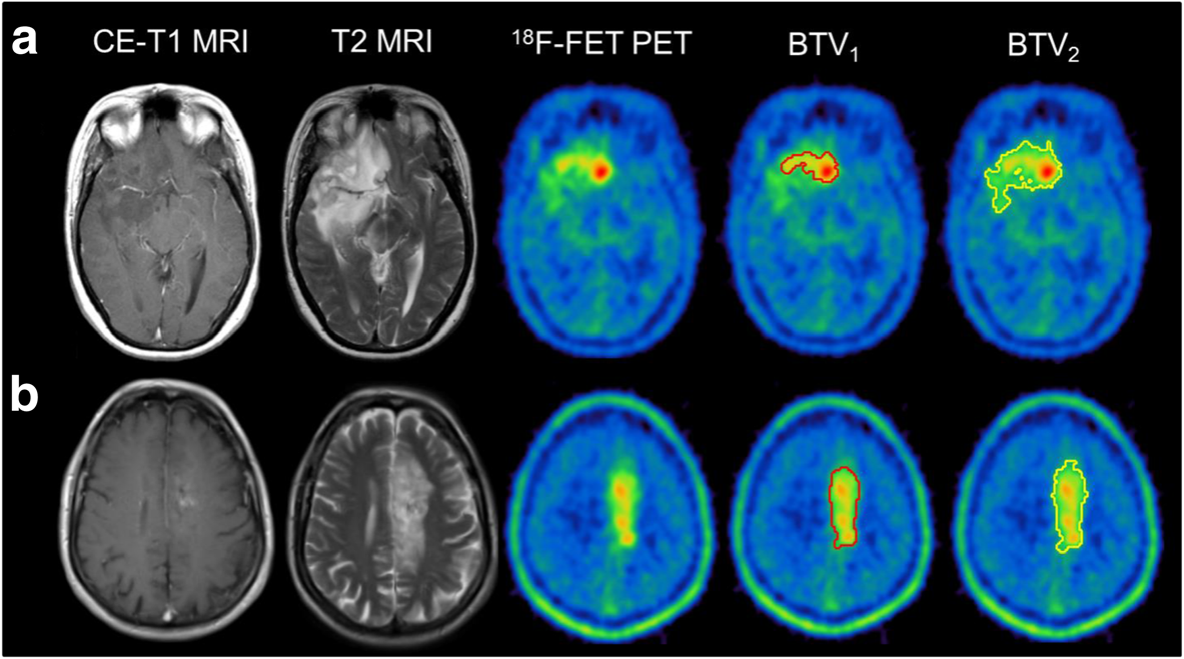
Influence of different background activity values on TBR_max_ and BTV values. **a** An example of a simple 2D-ROI assessment with measured background SUV values between 1.17–1.50: the resulting TBR_max_ ranges between 3.10 and 4.01 (ΔTBR_max_, 0.91; %ΔTBR, 29%), the BTV delineated with the calculated lowest threshold of SUV 1.87 is 45.89 ml, while BTV using the threshold with the highest background SUV (threshold SUV 2.40) results in a BTV with 9.91 ml only (ΔBTV, 35.98 ml; %ΔBTV, 78%). **b** The guided VOI assessment with a maximal background SUV range of 0.83–0.87 results in a small TBR range of 2.99–3.13 (ΔTBR_max_, 0.14; %ΔTBR_max_, 4%) and a BTV range of 20.53–22.63 ml (ΔBTV, 2.10 ml; %ΔBTV, 9%)

Although the overall variability might seem moderate at first sight, it is important to note that there are outliners up to a CoV of 8% when using the spherical approach. In clinical routine, this leads to an expectable difference of up to +8% as well as −8% of the background SUV. This would not only lead to substantially different TBR values but also to substantially different thresholds for the delineation of the BTV, which is commonly defined by 1.6 × background activity. For visualization, the maximal intra-reader as well as inter-reader differences and their consequences on the clinically important ^18^F-FET PET parameters are presented in clinical examples (see Fig. 4).

In the clinical routine, high differences regarding the TBR could substantially influence the conclusion of a PET scan and thereby hamper the diagnostic value, e.g., when used for differentiation of treatment-related changes and viable tumor [11–13]. In particular, distinct cutoff values for the evaluation of gliomas are used in clinical routine [3, 10], e.g., the cutoff TBR_mean_ ≥ 2.0 differentiates progressive disease from treatment-related changes with a high accuracy. This is also true for the BTV evaluation, e.g., when used for response-assessment during chemotherapy [7–9], where distinct changes of, e.g., 20% are considered as treatment response and vice versa. Additionally, besides the mere quantitative information of the volumetric measure, the spatial localization and extent of the tumor tissue, influenced by the background assessment, might also have an impact on the clinical workup, e.g., regarding radiotherapy planning [2, 24]. Nonetheless, the interfering factor can considerably be reduced as highlighted using the crescent-shaped VOI approach for background activity assessment.

This is especially the case since the application of the crescent-shaped VOI showed the lowest variabilities in the subgroups of the experienced and unexperienced readers in terms of both intra- and inter-reader variability. Additionally, the use of the detailed instructions for a guided VOI could additionally reduce the variability. It should be pointed out that possibly influencing factors, such as “non-neoplastic” lesions (e.g., major infarctions in MRI, cysts), should not be included in the background VOI.

Our results are of high interest with regard to multicenter comparisons and anticipated multicenter studies, which require a high degree of standardization in order to provide reproducible and reliable PET data in all sites performing amino acid imaging of brain tumors. Besides the standardization of ordinary influencing factors such as reconstruction algorithms and PET scanner type, it will, according to our results, be important to standardize the particular method of background activity assessment.

Although a software-based method had already been suggested for [^11^C]-methionine PET 10 years ago [21], it is essential to find a quick and unpretentious, but reliable approach without any need of further hardware or software solutions, which might be suitable for basic research applications, but surely hamper the clinical implementation. Within the commonly applied methods, the crescent-shaped VOI assessment showed the lowest variabilities even in unexperienced readers and the best results were reached with simple instructions for a guided VOI assessment. We therefore propose this method as possible and clinically applicable approach for methodological standardization, which is strongly needed, since the current RANO/EANO recommendations for the clinical use of PET imaging in gliomas, which were recognized very positively in the literature [25, 26], emphasized the implementations of amino acid PET in future prospective multicenter trials but pointed out that “[…] numerous studies differed in terms of methodology, which limits comparability of data and might eventually jeopardize acceptance in the clinical setting.” [1]. In the context of the anticipated standardized technical guidelines for glioma PET imaging procedures and recommendations by the EANM, EANO, and RANO [1], the use of a standardized approach for background activity assessment might be an important methodological landmark.

## Conclusions

Among the commonly used methods for the assessment of background activity in ^18^F-FET PET, the (guided) crescent-shaped VOI in the contralateral hemisphere reveals the most stable results and can substantially minimize both intra- and inter-reader variability. It represents a quick and easily applicable approach for the clinical routine, which might facilitate comprehensive methodological standardization of amino acid PET evaluation and strengthen its value in the clinical setting.

## References

[1] Albert NL, Weller M, Suchorska B, Galldiks N, Soffietti R, Kim MM (2016). Response Assessment in Neuro-Oncology working group and European Association for Neuro-Oncology recommendations for the clinical use of PET imaging in gliomas. Neuro Oncol.

[2] Niyazi M, Geisler J, Siefert A, Schwarz SB, Ganswindt U, Garny S (2011). FET–PET for malignant glioma treatment planning. Radiother Oncol.

[3] Unterrainer M, Schweisthal F, Suchorska B, Wenter V, Schmid-Tannwald C, Fendler WP, et al. Serial 18F-FET PET imaging of primarily 18F-FET-negative glioma—does it make sense? J Nucl Med. 2016:10.2967/jnumed.115.171033. 10.2967/jnumed.115.171033 27033893

[4] Jansen NL, Suchorska B, Wenter V, Eigenbrod S, Schmid-Tannwald C, Zwergal A (2014). Dynamic 18F-FET PET in newly diagnosed astrocytic low-grade glioma identifies high-risk patients. J Nucl Med.

[5] Jansen NL, Suchorska B, Wenter V, Schmid-Tannwald C, Todica A, Eigenbrod S (2015). Prognostic significance of dynamic 18F-FET PET in newly diagnosed astrocytic high-grade glioma. J Nucl Med.

[6] Galldiks N, Dunkl V, Stoffels G, Hutterer M, Rapp M, Sabel M (2015). Diagnosis of pseudoprogression in patients with glioblastoma using O-(2-[18F] fluoroethyl)-L-tyrosine PET. Eur J Nucl Med Mol Imaging.

[7] Galldiks N, Rapp M, Stoffels G, Fink GR, Shah NJ, Coenen HH (2013). Response assessment of bevacizumab in patients with recurrent malignant glioma using [18F] fluoroethyl-L-tyrosine PET in comparison to MRI. Eur J Nucl Med Mol Imaging.

[8] Hutterer M, Nowosielski M, Putzer D, Waitz D, Tinkhauser G, Kostron H (2011). O-(2-18F-fluoroethyl)-L-tyrosine PET predicts failure of antiangiogenic treatment in patients with recurrent high-grade glioma. J Nucl Med.

[9] Unterrainer M, Suchorska B, Biczok A, Bartenstein P, Kreth F-W, Albert N (2016). Value of 18F-FET PET for chemotherapy monitoring in non-contrast enhancing gliomas. J Nucl Med.

[10] Galldiks N, Stoffels G, Filss C, Rapp M, Blau T, Tscherpel C (2015). The use of dynamic O-(2-18F-fluoroethyl)-l-tyrosine PET in the diagnosis of patients with progressive and recurrent glioma. Neuro Oncol.

[11] Ceccon G, Lohmann P, Stoffels G, Judov N, Filss CP, Rapp M, et al. Dynamic O-(2-18F-fluoroethyl)-L-tyrosine positron emission tomography differentiates brain metastasis recurrence from radiation injury after radiotherapy. Neuro Oncol. 2017;19(2):281–8. 10.1093/neuonc/now149 PMC5463967 27471107

[12] Galldiks N, Stoffels G, Filss CP, Piroth MD, Sabel M, Ruge MI (2012). Role of O-(2-18F-fluoroethyl)-L-tyrosine PET for differentiation of local recurrent brain metastasis from radiation necrosis. J Nucl Med.

[13] Romagna A, Unterrainer M, Schmid-Tannwald C, Brendel M, Tonn J-C, Nachbichler SB (2016). Suspected recurrence of brain metastases after focused high dose radiotherapy: can [18 F] FET-PET overcome diagnostic uncertainties?. Radiat Oncol.

[14] Vander Borght T, Asenbaum S, Bartenstein P, Halldin C, Kapucu Ö, Van Laere K (2006). EANM procedure guidelines for brain tumour imaging using labelled amino acid analogues. Eur J Nucl Med Mol Imaging.

[15] Floeth FW, Pauleit D, Sabel M, Reifenberger G, Stoffels G, Stummer W (2006). 18F-FET PET differentiation of ring-enhancing brain lesions. J Nucl Med.

[16] Hutterer M, Nowosielski M, Putzer D, Jansen NL, Seiz M, Schocke M (2013). [18F]-fluoro-ethyl-l-tyrosine PET: a valuable diagnostic tool in neuro-oncology, but not all that glitters is glioma. Neuro Oncol.

[17] Langen KJ, Bartenstein P, Boecker H, Brust P, Coenen HH, Drzezga A (2011). PET- und SPECT-Untersuchungen von Hirntumoren mit radioaktiv markierten Aminosäuren. Nuklearmedizin.

[18] Lohmann P, Stoffels G, Ceccon G, Rapp M, Sabel M, Filss CP, et al. Radiation injury vs. recurrent brain metastasis: combining textural feature radiomics analysis and standard parameters may increase 18F-FET PET accuracy without dynamic scans. Eur Radiol. 2016. [Epub ahead of print]. 10.1007/s00330-016-4638-2 27853813

[19] Lohmann P, Herzog H, Rota Kops E, Stoffels G, Judov N, Filss C (2015). Dual-time-point O-(2-[18F]fluoroethyl)-L-tyrosine PET for grading of cerebral gliomas. Eur Radiol.

[20] Munck af Rosenschold P, Costa J, Engelholm SA, Lundemann MJ, Law I, Ohlhues L (2015). Impact of [18F]-fluoro-ethyl-tyrosine PET imaging on target definition for radiation therapy of high-grade glioma. Neuro Oncol.

[21] Coope DJ, Čížek J, Eggers C, Vollmar S, Heiss W-D, Herholz K (2007). Evaluation of primary brain tumors using 11C-methionine PET with reference to a normal methionine uptake map. J Nucl Med.

[22] Jansen NL, Graute V, Armbruster L, Suchorska B, Lutz J, Eigenbrod S (2012). MRI-suspected low-grade glioma: is there a need to perform dynamic FET PET?. Eur J Nucl Med Mol Imaging.

[23] Pauleit D, Floeth F, Hamacher K, Riemenschneider MJ, Reifenberger G, Müller H-W (2005). O-(2-[18F] fluoroethyl)-L-tyrosine PET combined with MRI improves the diagnostic assessment of cerebral gliomas. Brain.

[24] Piroth MD, Galldiks N, Pinkawa M, Holy R, Stoffels G, Ermert J (2016). Relapse patterns after radiochemotherapy of glioblastoma with FET PET-guided boost irradiation and simulation to optimize radiation target volume. Radiat Oncol.

[25] Langen K-J, Watts C (2016). Neuro-oncology: amino acid PET for brain tumours [mdash] ready for the clinic?. Nat Rev Neurol.

[26] Watts C, Langen K-J (2016). PET imaging in glioma: is it time for mainstream practice?. Neuro Oncol.

